# An acoustical dataset of storytelling and story reading in Jordanian Arabic

**DOI:** 10.1016/j.dib.2026.112875

**Published:** 2026-05-19

**Authors:** Mohammad Abuoudeh

**Affiliations:** Department of Language and Linguistics, Al-Hussein Bin Talal University, Ma'an p. B (20) 71111, Jordan

**Keywords:** Speaking style, Continuous speech, Jordanian arabic, Speech corpus

## Abstract

This article presents a phonetic corpus that captures phonetic and lexical variation across two speaking styles in Jordanian Arabic. The database was constructed based on the voluntary participation of 109 native speakers (64 males and 45 females) from various governorates of Jordan. All participants were university students ranging in age from 18 to 27. The corpus consists of two tasks: storytelling and story reading, both based on an adapted Jordanian Arabic version of “*Laylaa Witheeb”* (*Little Red Riding Hood*). The adaptation was prepared by the experimenter, and participants were instructed to use their native dialect. In the first task, participants read the story from a computer screen, while in the second, they retold the story without referring to the text, thereby aiming to elicit a natural storytelling context. The collected speech data were transcribed in Arabic orthography, then transliterated using a phonetic transcription system, and finally segmented by forced alignment. Subsequent manual verification was performed in the Praat software, resulting in a phonetic database ready for research applications. The development of this corpus addresses the lack of Jordanian Arabic speech resources, facilitating comparative and quantitative linguistic analyses.

Specifications Table

**Table:** 

Subject	Social Sciences
Specific subject area	Acoustic database containing the recordings of 109 speakers producing story reading and storytelling tasks for spectral and linguistic analysis.
Type of data	Automatically force-aligned and manual corrected audio files (.wav) TextGrid with three Tiers Arabic orthography transcription for both tasks (.docx) Phonetic transcription for both tasks (.txt) Metadata table (.ods)
Data collection	In a quiet room at the College of Art, 109 Jordanian native speakers (undergraduate students) were recorded reading and then telling a short story. Sennheiser e945 microphone connected to ZOOM H8 recorder was placed at a distance of 15 cm from the speakers’ mouth (44.1 kHz/16-bit). In the reading task, speakers read the text written in undiacritized Arabic orthography from a computer screen.
Data source location	Al-Hussein Bin Talal University, Ma'an, Jordan
Data accessibility	Repository name: Mendeley Data identification number: doi: 10.17632/v9n7g7ns49.1 Direct URL to data: https://data.mendeley.com/datasets/v9n7g7ns49/1
Related research article	None

## 1 Value of the Data

• The dataset represents a large number of high quality recordings of Jordanian Arabic dialects in two different speech production situations (story-reading and storytelling).
• All recordings have been automatically forced-aligned, and then verified and corrected manually.
• The dataset can be used in many different studies in linguistics and sociolinguistics. For instance, it can be used to compare differences in phonetic phenomena in both speaking styles across gender, dialect, sociocultural backgrounds. In addition, it can be used for phonological, syntactic, and semantic studies as the transcription of the texts for both reading and storytelling is provided in both Arabic and phonetic scripts.
• The dataset can be divided and used exclusively as a reading subset or as a storytelling subset.

## 2 Background

Building this database was originally motivated by the intention to study the impact of speaking style shifts on the spectral and temporal properties of long and short vowels in Jordanian Arabic. In fact, the storytelling style is considered a high cognitive load task demanding memory retrieval, lexical access, and conceptualisation processes (which are time consuming). In contrast, the reading style is considered a low cognitive load task requiring no memory retrieval or conceptualisation [1,2]. These situations create two interesting temporal and tensional conditions for the motor control. In the first situation, motor control seeks to adapt the articulatory gesture to the lack of time, while in the second, the motor control has no need to compromise to achieve its targets.

## 3 Data Description

The database consists of acoustic recordings from 109 speakers, the Arabic and phonetic transcriptions of these recordings, Praat segmentation files, a metadata table with the information about all speakers, and a Praat script to extract temporal and spectral information of the phonemes. The data is organized according to the place of residence of each speaker. It includes a total of 13 folders: 11 folders named after the Jordanian cities, as well as the central and southern Bedouin folders (*e.g.*, Amman, Ma’an, Bedouin_south, etc.). Inside each of these folders, there are individual speaker folders. The name of each speaker’s folder consists of the speaker’s initials (to preserve the speaker’s anonymity), a serial number (from 1 to 109), and the speaker’s gender (F for female and M for male), for example, BA31F. Inside each speaker’s folder, there are the following 8 files:

1. The recording of the reading task in .wav format, named as follows: [speaker’s folder name]_Laylawitheeb_read.
2. The recording of the storytelling task in .wav format, named as follows: [speaker’s folder name]_Laylawitheeb_told.
3. The Arabic orthographic transcription text of the reading task in .docx format, named after the corresponding recording file with the suffix Arabic_transcription.
4. The Arabic orthographic transcription text of the storytelling task in .docx format, named after the corresponding recording file with the suffix Arabic_transcription.
5. The ATR transliteration text (see the following section) of the reading task in .txt format, named after the corresponding recording file.
6. The ATR transliteration text of the storytelling task in .txt format, named after the corresponding recording file.
7. The Praat segmentation file of the reading task in .TextGrid format, named after the corresponding recording file.
8. The Praat segmentation file of the storytelling task in .TextGrid format, named after the corresponding recording file.

### 3.1 The metadata file

The metadata file contains the speakers’ information in .ods format and is named ‘Speaker_info’. All the information was gathered before the recording session and includes the following: the speaker’s ID (*e.g.*, JS1M), sex, region, dialect, field of study, place of birth, father’s occupation, mother’s occupation, father’s dialect, mother’s dialect, father’s place of birth, and mother’s place of birth. This information allows for sociocultural, dialectological, and gender studies.

### 3.2 The Praat script file

A Praat script is provided in the database to extract the spectral and temporal information, facilitating acoustical studies on this dataset. This script allows for the extraction of segment and word durations, the frequencies of the first three formants (F1, F2, and F3), and the fundamental frequency (f0). The *Burg* extraction algorithm (LPC analysis *via* autocorrelation) is used with an analysis window of 0.025 s and a time step of 0.01 s. The formant extraction settings can be adapted according to the speaker’s gender (5500 Hz for females and 5000 Hz for males). Similarly, the f0 extraction range can be adapted according to the gender (100–500 Hz for females and 75–300 Hz for males). The extracted information is saved in a .csv file. This script can be adapted for specific studies by adding, modifying, or deleting commands.

## 4 Experimental Design, Materials and Methods

### 4.1 Speakers

A total of 109 native speakers of Jordanian Arabic (64 males and 45 females) participated voluntarily in this experiment. The recordings were conducted between 2023 and 2024, and all participants were undergraduate students at Al-Hussein Bin Talal University and were ages between 18 and 27 years old (mean = 21) at the time of recording. All speakers reported that they did not suffer from any language disorders. They came from All Jordanian governorates (except the Jerash governorate, as no students from this region participated). The participants from villages in the centeral or the southern region and declared that they speak Bedouin dialect were classified as Bedouin center and Bedouin south. The Table 1 presents the distribution of the participants by governorate, gender, and mean age.

**Table 1:** Distribution of speakers by region, indicating the number of male and female speakers, along with their mean age (and standard deviation).

Region	Male	Female	Total	Age
Amman	7	8	15	21(1.1)
Bedouin south	7	5	12	22(2.1)
Maan	6	5	11	21(2.2)
Zarqa	6	5	11	20(1)
Karak	5	5	10	20(1)
Tafilah	5	4	9	21(1.4)
Mafraq	5	3	8	21(1)
Salt	4	4	8	21(1.3)
Irbid	5	2	7	20(1.2)
Aqaba	5	2	7	20(1.3)
Bedouin center	5	0	5	23(2)
Madaba	2	2	4	21(1.3)
Ajlun	2	0	2	22(1.4)
Total	64	45	109	21(1.6)

Before the beginning of the recordings, the participants were briefly informed about the purpose of the experiment and signed a consent form indicating that their recordings would be used for research purposes, that their anonymity would be guaranteed, and that they had the right to withdraw from the experiment at any time. Participants were also asked to complete a questionnaire providing the following information: name, age, place of residence, place of birth, place of birth of parents, university major, parents’ occupations, the dialect they believe they speak, and the dialect they believe their parents speak. These details provide valuable sociocultural information that can be used in sociolinguistic studies. It is interesting to note that regarding the question on the spoken dialect, some participants stated that they did not know which dialect they spoke, or they reported that they spoke the Jordanian dialect. In these cases, the experimenter asked them to specify the dialect they believed they spoke in terms of regional dialects in Jordan, such as Ma’ani, Bedouin, Karaki, etc. All this collected information was anonymized and saved in an .ods file.

### 4.2 Linguistic material

The recorded corpus is based on the story of the *Little Red Riding Hood* (“Laylaa witheeb” in Arabic), written in Arabic orthography without vocalization and adapted into a Jordanian Arabic version by the experimenter. It is important to note that this story is popular in Jordan, and all the participants reported being familiar with it. The choice of a well-known and popular story was intended to facilitate the storytelling task.

### 4.3 Procedure

Participants were asked to read the Little Red Riding Hood story from a text displayed on a computer screen (the reading task). Subsequently, they were asked to retell the same story from memory (the storytelling task). Before recording the storytelling task, participants were allowed to read the story silently to prepare for the task if they judged it necessary. Participants were reminded to read and tell the story in their own dialect and not in Standard Arabic. The instruction given to participants was: “Read, and then retell the story as if you were reading or telling it to one of your family members”. Participants were permitted to replace certain words in the text that they did not use in their own dialect. For example, the word for ‘’my grandmother” could have three alternatives depending on the dialect: /jiddatiː/ vs. /sittiː/ vs. /teːtaː/.

The experiment was conducted in a quiet room at the Faculty of Arts in Al-Hussein Bin Talal University. The equipment used for the recordings consisted of a Sennheiser e945 microphone connected to a Zoom H8 recorder. The microphone was mounted on a desktop microphone stand and was placed approximately 15 cm from the participant’s mouth. The recording parameters were set to a 44,100 Hz sampling rate with a 32-bit resolution in mono. The duration of the reading task recording was approximately two minutes, while the storytelling recording duration varied by participant, ranging between one and two minutes in most cases. This difference between tasks is mainly due to the fact that speakers omitted phrases and summarized the story with their own words in the storytelling task.

### 4.4 Transcription and segmentation

**Fig. 1:**
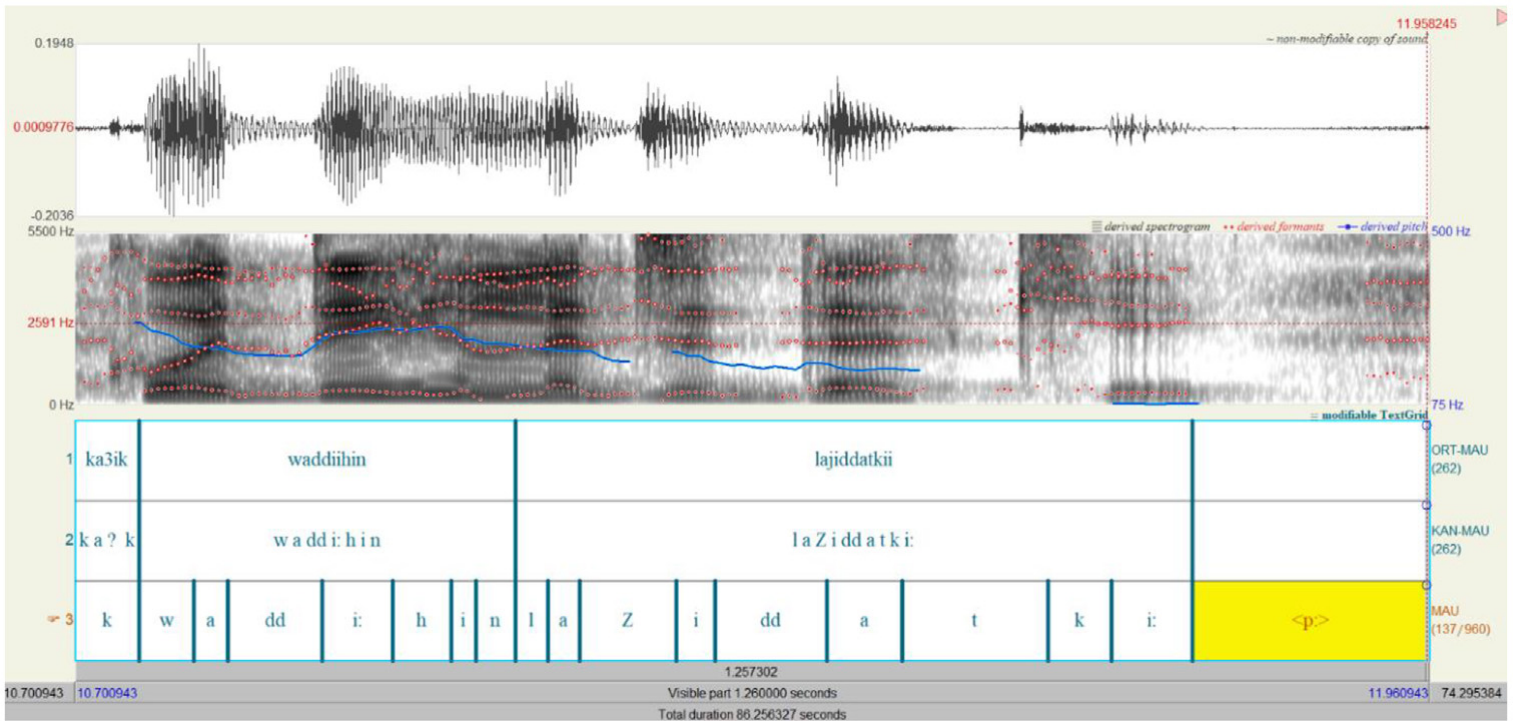
A screen shot of a Praat window showing the transcription and the segmentation generated by the forced alignment, and subsequently corrected manually for the phrase “waddiihin lajiddatkii” (send them to your grandmother) by a female speaker from karak.

The audio files from the two tasks were automatically transcribed in Arabic orthography via the Transcribe function in Microsoft Word. This transcription was then manually verified and corrected by the experimenter. The transcribed text in Arabic orthography was then transliterated using the new Arabic transliteration system, the “ATR convention” [3]. The Arabic orthographic text was first converted to the Latin alphabet via a website (https://mylanguages.org/arabic_romanization.php) and then verified and corrected manually by the experimenter. The text transcribed using the ATR convention allows for the forced alignment by using the *ArabicWebMAUSBasic* service from the WebMAUS^1^ website, which provides a segmented .TextGrid files [3,4]. The results from the forced alignment (.TextGrid files) were manually verified and corrected by the experimenter using Praat [5]. The .TextGrid files contain three tiers that are automatically generated and named (ORT-MAU, KAN-MAU, and MAU) by the *ArabicWebMAUSBasic* service (Fig. 1). The first tier, ORT-MAU, contains the segmentation of the words transcribed in ATR. The second tier, KAN-MAU, contains the segmentation of the word transcribed in X-SAMPA. Finally, the third tier, MAU, contains the phoneme-level segmentation in X-SAMPA.

Regarding the manual correction of the force-aligned segmentation, the boundaries (onset and offset) of each word were determined based on the acoustic characteristics of the initial and final phonemes. For words beginning with a voiced stop, the beginning of the voicing bar was considered as the beginning of the word/phoneme. For word-initial voiceless stops directly following a preceding word (without a significant pause), the offset of the final phoneme of the preceding word was considered as the onset boundary. However, when a word-initial voiceless stop is preceded by a long pause, the exact onset of the silent closure is acoustically unrecoverable from a spectrogram [6]. In this case, a fixed closure duration of 30 ms prior to the release burst was assigned. This choice was motivated by perceptual constraints on speech processing, where a minimal silent closure of 20 to 30 ms is required for the perception of voiceless stops (for example, [7]). Concerning the words starting with other phoneme classes, the beginning of the voicing for the voiced phonemes and the beginning of the frication noise for the fricatives indicated the beginning of the word/phoneme. The final boundary of the word was determined by the end of the voicing for the voiced phonemes, the end of the release burst for stops, and the end of the frication noise for fricatives. It is worth noting that words produced with one or more errors were excluded from the segmentation. Furthermore, filled pauses (such as eeh, aaa) and the lengthening of final vowels related to hesitation were segmented. Documenting this is important for future users, as the atypical durations of these hesitation vowels could skew statistical analyses of vowel duration. In such cases, users can simply filter out the data related to these vowels during data analysis.

## Limitations

During the recording phase, the experimenter recorded the participants coming from different Jordanian regions without controlling for an equal number of participants from each region (as recording was dependant on volunteer participation). Consequently, the distribution of participants per region is not balanced, which could influence studies comparing specific regional dialects in Jordan. This limitation can be resolved by grouping the participants into the three traditional dialectal categories: Urban, Rural, and Bedouin.

## Ethics Statement

All participants signed a written informed consent form to participate in the study. The study was conducted in strict compliance with the principles of the Helsinki Declaration. According to the research guidelines of the funding institution, Al-Hussein Bin Talal University, formal approval from an ethics review committee was not required for this type of research at the time of data collection.

## CRediT Author Statement

**Mohammad Abuoudeh:** Conceptualization, Methodology, Validation, Formal analysis, Investigation, Resources, Data curation, Writing –original draft, Project administration, Funding acquisition.

## Data Availability

Mendeley DataLayla Witheeb: Jordanian Arabic Acoustic Dataset (Original data)

## Footnotes

1 https://clarin.phonetik.uni-muenchen.de/BASWebServices/interface/WebMAUSBasic
